# Supplementary material for: Macromolecular Crowding Increases the Affinity of the PHD of ING4 for the Histone H3K4me3 Mark
Source: Biomolecules. 2020 Feb 4;10(2):234. doi: 10.3390/biom10020234 (PMC7072245; doi:10.3390/biom10020234)
Supplement: Supplementary file 1 [file biomolecules-10-00234-s001.pdf]

Supplementary Information for

Macromolecular crowding increases the affinity of the PHD of ING4 for the histone H3K4me3 mark

Alicia Palacios and Francisco J. Blanco*

CIC bioGUNE, Parque Tecnológico de Bizkaia, Edificio 800, 48160 Derio, Spain

*Corresponding author. E-mail: fblanco@cicbiogune.es

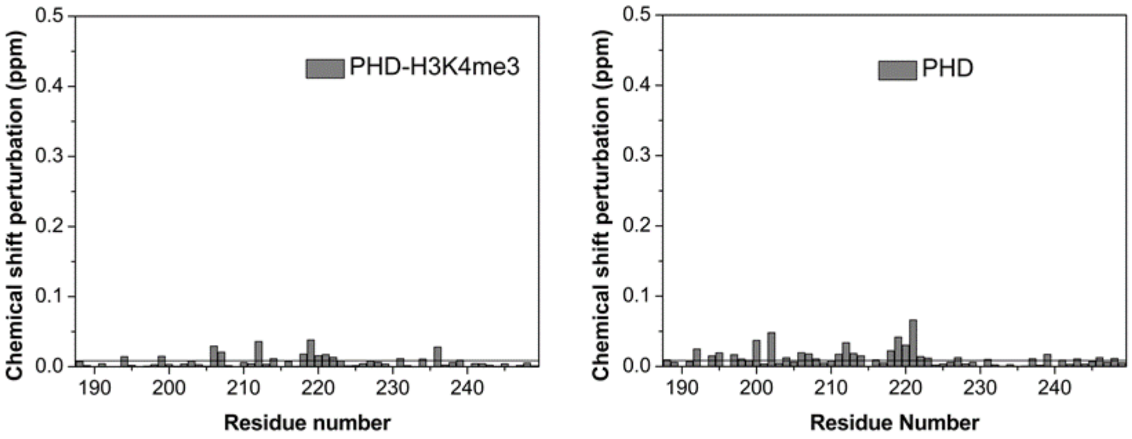

Supplementary Figure 1. Effect of 15% Ficoll 70 on the backbone amide chemical shifts of ING4-PHD bound to H3K4me3 peptide (left) or free (right). The bar plots show the CSP observed for each residue in ^{1}H-^{15}N HSQC spectra of 50 µM PHD in the presence or absence of a 4-fold molar excess of H3K4me3 in 20 mM sodium phosphate pH 6.5, 50 mM NaCl, 1 mM perdeuterated dithiothreitol, 15% Ficoll 70, 5% $^{2}H_2O$, and 0.01% NaN_3 at 25 °C. The estimated experimental error is 0.008 ppm and is indicated with a horizontal line.

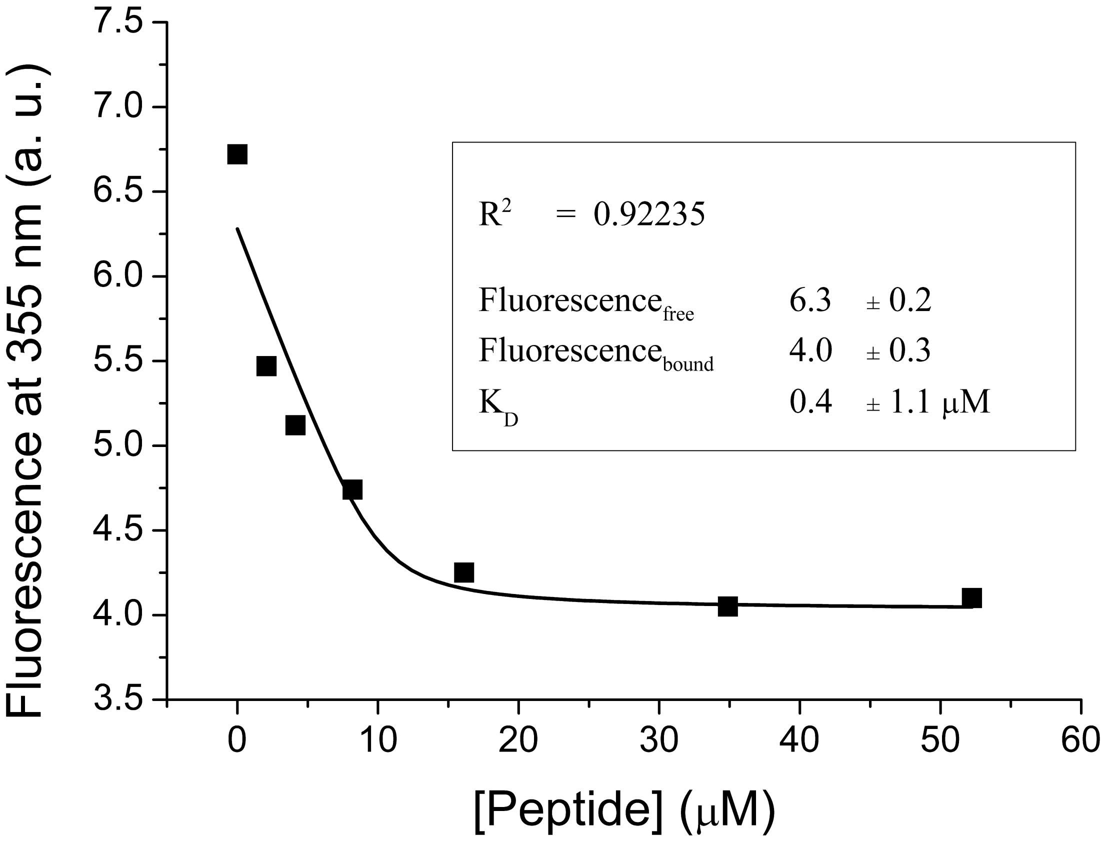

Supplementary Figure 2. Binding isotherm of H3K4me3 peptide to the PHD finger of ING4 as measured by the changes in the intrinsic fluorescence of the PHD protein at 25 °C. The solid line is the fitting to an equilibrium with a single set of binding sites. The Adjustable parameters are indicated with their corresponding errors. The protein concentration was 10 μM in 20 mM sodium phosphate pH 6.5, 150 mM NaCl, 1 mM dithiothreitol, 15% Ficoll 70. The data were measured on a Perkin Elmer LS55B using an excitation wave length of 280 nm and 7 nm slit widths.
